# A pin-based probe for electronic moisture meters to determine moisture content in a single wheat kernel

**DOI:** 10.1186/s13007-024-01194-3

**Published:** 2024-06-10

**Authors:** Jatinder S. Sangha, Brad Meyer, Yuefeng Ruan, Richard D. Cuthbert, Ron Knox, Gaozhi Xiao

**Affiliations:** 1 Swift Current Research and Development Centre, Agriculture and Agri-Food Canada, Swift Current, Saskatchewan, S9H 3X2 Canada; 2 National Research Council, Ottawa, ON K1A 0R6 Canada

**Keywords:** Kernel moisture, Moisture probe, Physiological maturity

## Abstract

**Background:**

Optimum moisture in straw and grain at maturity is important for timely harvesting of wheat. Grain harvested at the right time has reduced chance of being affected by adverse weather conditions which is important to maintain grain quality and end use functionality. Wheat varieties with a short dry down period could help in timely harvest of the crop. However, measuring single kernel moisture in wheat and other small grain crops is a phenotyping bottleneck which requires characterising moisture content of the developing kernel at physiological maturity.

**Results:**

Here we report developing a pin-based probe to detect moisture in a developing wheat kernel required for determining physiological maturity. An in-house designed pin-based probe was used with different commercially available electronic moisture meters to assess the moisture content of the individual kernels in spikes with high accuracy (R^2^ = 0.73 to 0.94, *P* < 0.001) compared with a reference method of oven drying. The average moisture values varied among different electronic moisture meters and the oven-dry method and differences in values were minimized at low kernel moisture content (< 50%). The single kernel moisture probe was evaluated in the field to predict the physiological maturity in wheat using 38% moisture content as the reference and visible notes on kernel stage.

**Conclusion:**

The pin-based moisture probe is a reliable tool for wheat physiologists and breeders to conveniently and accurately measure moisture content in developing grain that will aid in identifying wheat germplasm with fast dry-down characteristics.

## Background

Trouble-free harvesting of small grain cereal crops requires an optimum level of moisture in straw and grain. Grain harvested at the right time has a reduced chance of being affected by adverse weather conditions such as rain, temperature, sun, and wind (Brooking [4]; Calderini et al. [5]; Nielsen [20]). Damp kernels can lead to several quality issues affecting end use functionality of the grain, development of mold, and lower market grade (Preston et al. [21]). The time for a crop to reach the required moisture level for harvesting is often challenging, especially on the Canadian prairies, due to the short growing season and unpredictable weather conditions. Dampness at harvest increases the urge for accelerated drying of the straw and grain through applying pre-harvest management practices such as swathing or a desiccant. These practices if not timed properly and as recommended, could impact yield and quality and increase the overall production costs (Carlson et al. [6]).

A solution for timely harvest of cereal crops is to develop varieties with an inherent short dry down period (Calderini et al. [5]). Such variety development needs large scale germplasm screening requiring continuous visible observations during the grain development period and measuring water dynamics of the kernel to identify physiological maturity, followed by selecting germplasm which takes the least time from physiological maturity to harvest maturity (Cross [8]; Mahesha et al. [17]). Monitoring kernel moisture before the grain dries down to 30% or less moisture is also important to reduce the need of spraying a desiccant, eliminating the risks of chemical residues that could deteriorate the quality and market value of the grain (Keep it Clean.ca).

Generally, to know the grain moisture content at harvest requires bulking a large number of plants from the field, followed by threshing the grain and then using commercial moisture measuring tools for moisture analysis (Clarke et al. [7]). These devices determine moisture content based on various parameters such as temperature, fresh and dry weight, electrical properties, and biophysical characteristics of the sample (Batista et al. [1]; Jones et al. [13]). Although, such instruments are calibrated against a standard method, such as an oven dry method (ISO t712: [11]), predicted average moisture of the bulk grain sample could vary depending on the kernel maturity that affects the moisture content. Moreover, in breeding programs, a large number of different lines are being handled each with a small grain sample processed for trait screening. It also requires sufficient accuracy and efficiency in moisture detection so as to confidently screen more samples in a limited time frame. As such, the common available methods of grain moisture determination will not be convenient in breeding due to the time and labor involved in the harvesting, cleaning, weighing and drying the seed samples.

Grain moisture measurement technology is highly advanced and methods such as microwave resonator (Kraszewski et al. [15]), open-ended waveguide technique with type-N microwave connector (Zhang et al. [24]), and near infrared spectroscopy (Watanabe et al. [23]) are developed. Electrical resistance methods for determining grain moisture content have existed ever since the beginning of the 19th century (Briggs [3]). With the advancement of science and technology, new devices that are portable, light weight, and low cost are available for use in a number of agriculture crops such as corn (Reid et al. [22]; Zhang et al. [25]), wheat (Zhang et al. [24]), peanut (Nelson et al. [19]; Dowell and Lamb [9]), and wood trees such as pine (Watanabe et al. [23]). A few studies report moisture meters for single kernels (Kang et al. [14]; Dowell and Lamb [9]; Kraszewski et al. [15]; Reid et al. [22]; Li et al. [16]). However, most of these devices are used for a narrow moisture range that is less than 40% moisture content. Moreover, no device is presently available to determine moisture content of a single kernel in a wheat spike, a requirement for studying moisture dynamics of the developing kernels and detecting the stage of physiological maturity of a plant.

The use of a moisture meter for determining grain moisture content has been well emphasized in breeding programs (Reid et al. [22]; Martinez-Feria et al. [18]). The significance of a portable and reliable moisture meter for single wheat kernel relies on the application potential for determining physiological maturity of multiple genotypes in a short time and identifying dry-down characteristics. Apart from its application in research, a single kernel moisture meter will be useful to growers for determining variability in moisture content in wheat spike to decide when to harvest the crop and avoid grain yield and quality loss from residual moisture in the grain due to not achieving physiological maturity in time.

In the absence of a moisture meter for application in the field with small grain cereals, this study was pursued to develop a low cost portable pin-based probe that is compatible with different electronic moisture meters to detect moisture in developing wheat kernels. Proof of concept of monitoring grain dry-down through the application of the optimum pin-based probe and a moisture meter was also undertaken.

## Results and discussion

A pin probe is developed to determine moisture content of a single kernel in a wheat spike. Using different moisture meters that are built on the principles of electrical conductivity and resistance between electrodes, this wheat kernel specific pin probe was designed for applying in field grown wheat to predict the moisture content of the kernel in the spike. The pin probe is suitable for studying moisture dynamics of the developing kernels and detecting the stage of physiological maturity in wheat.

### Designing a single kernel probe

**Fig. 1:**
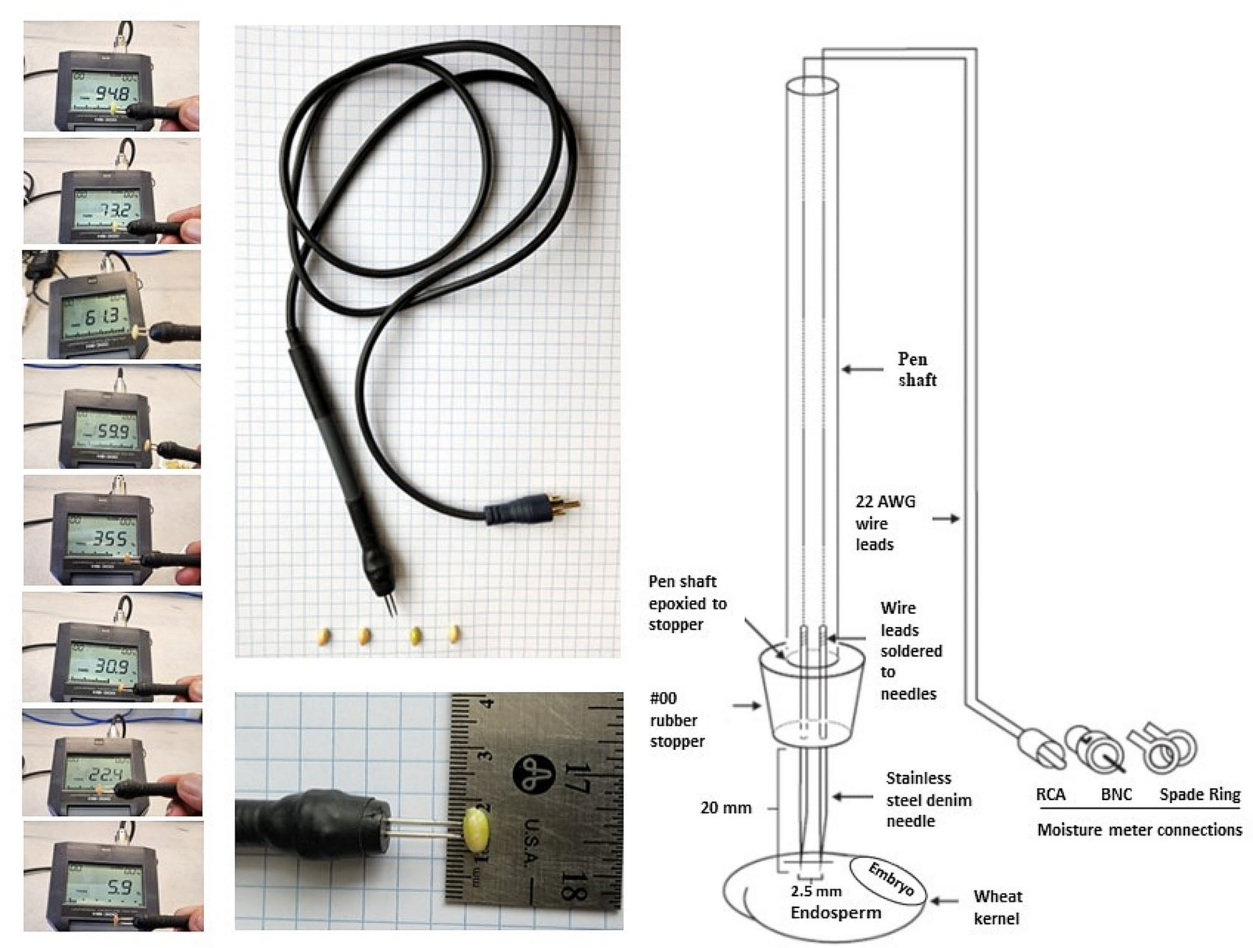
Design of the single kernel moisture probe for wheat application

To develop proof-of-concept single kernel moisture detection probe that could puncture through the glume into a small (4–6 mm) wheat kernel in the spikelet, a pair of fine, sharp, and highly conductive metal pins was needed so that there would be minimal loss of electrical signal to the moisture reader. Strong metal pins were also required to prevent breaking or bending of the pin electrodes while pushing through sample material, such as a hard maturing kernel. Multiple probes were made using the same design and exchangeable adaptors for attaching with the meters. The metal pins embedded in a solid nonconductive rubber block was done to prevent signal loss (Briggs [3]; Nelson et al. [19]; Reid et al. [22]). A high quality stranded and insulated 22 AWG wire connecting the probe to the moisture reader was selected for its balance of durability for field use and high solderability allowing for good signal transmission to the moisture meter. A pictorial description of the pin-based probe is given in the Fig. 1.

The denim cloth sewing needles as pin electrodes used in the study were able to transmit the signal effectively as observed by using a standard moisture calibration disk of 16%. A distance of 2.5 mm between needle tips was purposely kept consistent as it was determined to optimally accommodate varying sizes of wheat kernels. Tips closer than 2.5 mm would make assembly difficult (needle insertion, soldering), while tips wider than 2.5 mm may not be insertable into smaller wheat kernels. The size and distance between the pin electrodes (2.5 mm) were suitable to pierce through the longitudinal section of the wheat kernel, and was compatible for use with a kernel having a minimum length of 4 mm. While gap width between the electrodes could influence on the sensor’s performance for moisture detection (Li et al. [16]), in our case, a slight variation in the distance between two pin electrodes set beyond 2.5 mm did not change the readability of the moisture content when tested repeatedly on different kernels and tested with a calibration disk. The presence of dew drops on the spike in the field may cause change in conductivity and affecting the results, this can be avoided by collecting data later in the afternoon when dew has evaporated.

### Detecting single kernel moisture content

The results of all four moisture measuring devices (Table 1) showed a significant positive relationship of moisture content with that of the oven dry method (Table 2). The relatively strong correlations ranging from *r* = 0.83 (MMH800) to *r* = 0.97 (HB300) between oven dry method and moisture content measured with the commercial devices were highly positive and significant (*P* < 0.001). The results also show a high coefficient of determination (R^2^) for all the moisture reading devices when regressed with the oven dry method. The R^2^ was calculated with linear (LR) and non-linear regression (NLR) and it was lowest for MT808C (LR = 0.73/NLR = 0.79) and it was highest for HB300 (LR = 0.94/ NLR = 0.94), whereas the R^2^ values for MMH800 (LR = 0.76/ NLR = 0.82) and MR55 (LR = 0.80/ NLR = 0.85) were intermediate to the other two devices. The accuracy of MMH808 may have been influenced by the limitation of the instrument not to make readings above 50% moisture. Based on correlation and coefficient of determination, the HB300 was the best performing device.

**Table 1:** Pin-based commercially available moisture readers used with single kernel moisture probe

Moisture reader	Model	Manufacturer	Uses	Moisture reading range
Moisture meter with corn probe	MT808C	Electrophysics, Canada	> 200 wood species, corn	4-100%, different calibrated groups
Universal Moisture tester	HB300	Kett, USA	Wood, concrete, grain, programmable	0–99%, user defined calibration
Pin Moisture Meter	MR55	FLIR, Canada	11 material groups, including timbers, drywall, and concrete	7–99%, four calibrated groups
Moisture meter	MMH800	General Tools, USA	lumber, drywall, subfloors, soil, paper, and powders	50% maximum, two calibrated groups

**Table 2:** The accuracy of the moisture measurements of single wheat kernels by different commercial moisture meters attached to an in-house developed single kernel probe by using correlation and regression of meter measurements against oven dry measurements of moisture

Moisture meter	Correlation coefficient (*r*) (Pearson’s/ Spearman’s)	Coefficient of determination (*R*^2)^	Regression equation	SEP* (%)
MT808C	0.86***	0.73	y = 0.416x + 16.28	9.87
	0.87	0.79	y = -0.0099 × ^2^ + 1.1999x + 1.8721	
MR55	0.89***	0.80	y = 0.3915x + 14.759	13.74
	0.90	0.85	y = -0.0064 × ^2^ + 0.9139x + 5.3974	
MMH800	0.87***	0.76	y = 0.5687x + 10.347	6.80
	0.83	0.82	y = -0.0135 × ^2^ + 1.4113x − 1.3836	
HB300	0.97***	0.94	y = 0.9728x − 0.1729	3.73
	0.97	0.94	y = -0.0014 × ^2^ + 1.0726x − 1.6177	

*SEP = Standard error of performance (prediction)

*** Highly significant (*p* < 0.001)

**Fig. 2:**
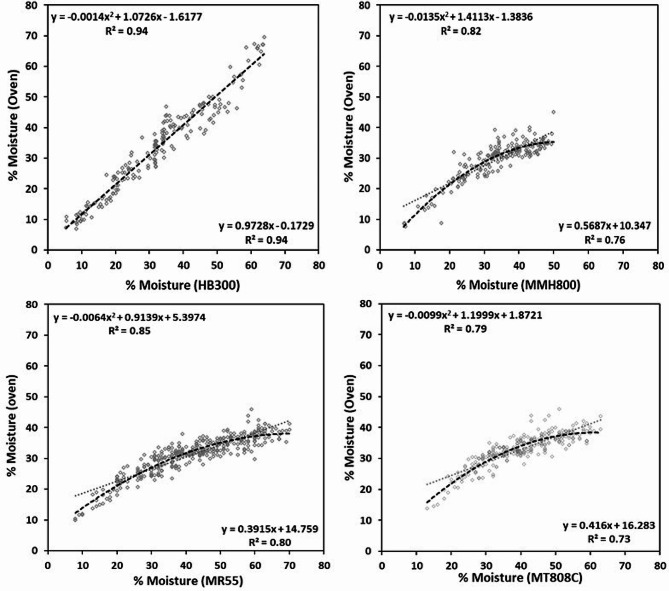
Relationship of oven method and commercial moisture meters equipped with a single kernel moisture probe for detecting moisture content (%) in wheat

The standard error of performance (SEP) values in Table 2 demonstrate the ability of the moisture probe with each device to reliably measure moisture content in kernels (Zhang et al. [24]). The SEP results for each device in the table shows that the SEP of 3.73 was the lowest for the HB300 device when compared with other three devices, with the highest SEP of 13.74 recorded for MR55. The low SEP, therefore, also indicated that the HB300 is better than other moisture meters due to the option to define the calibration for different tests. This calibration option was missing with other devices that are equipped with in-built calibrated models for moisture determination in various types of samples. Scatter plots of oven dry moisture measurements against electronic device measurements, along with regression equations, are given in Fig. 2 showing a good linear relationship and a non-linear (2nd degree polynomial) relationship across the different moisture determining devices. In comparison to the other three instruments, the scatter plot for MMH800 demonstrates the moisture measurement limitation by the device at 50% moisture.

A number of methods are available to determine the moisture content in grain, each having variability in reading the moisture content depending on conditions in which several environmental factors can be at play. Compared to our SEP results, Zhang et al. [24] show a higher SEP with multiple methods used for detecting moisture content, however, those moisture rates were well below 50% moisture content whereas the moisture content for this study ranged from 15% (mature kernel) to 70% (approximately the stage between early and mid-dough). In accordance to many other reports, we also noticed a departure in moisture detection accuracy of electronic devices compared to a gravimetric reference method when testing the early dough stage of the developing kernel with moisture content greater than 70%. However, as the kernel tends to mature, moisture content decreases and dry matter including starch, protein, minerals etc. accumulates. At this stage, variability in moisture content is reduced and detection accuracy improves. At higher moisture, these instruments are prone to inaccurate predictions and give a high % moisture reading when compared to the oven dry method. Therefore, we used an upper limit of 70% moisture for these instruments to improve the prediction accuracy of the moisture meters using the single kernel moisture probe. Our protocol covers a wide range of kernel moisture, much higher than most other reports, suggesting good detection capability of the probe we designed for wheat kernels.

As indicated by the SEP values in Table 2, no doubt, there is variation in the detection of the kernel moisture content between meters, which includes variation inherent in each of the commercially available device. The setting that we used for moisture detection is based on the option available for plant material, with exception of HB300. For example, the MMH800 is limited to a 50% moisture detection as an upper limit. On the other hand, HB300 is more advanced meter being capable of calibrations by the user according to temperature conditions of the application site when compared to the other devices with in-built calibrations set by the manufacturer. Nevertheless, the device HB300 was found more accurate in determining the moisture content with low SEP, a high R^2^ value and moisture prediction values closest to the oven dry method used for reference moisture content.

### Field application of single kernel pin-based probe in wheat

**Fig. 3:**
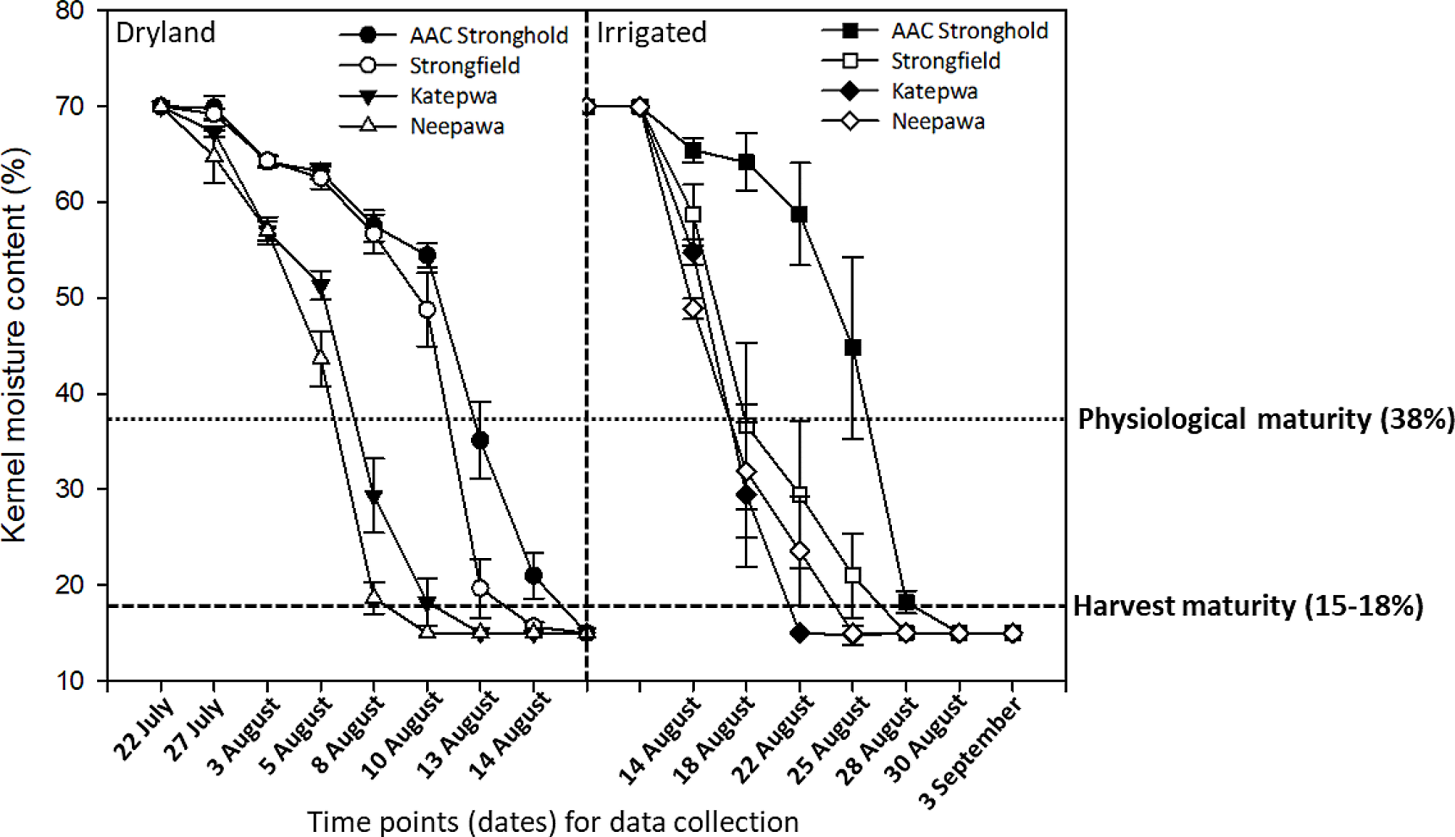
Application of single kernel pin probe with a commercial moisture meter for determining moisture at different time intervals in four wheat varieties, AAC Stronghold, Strongfield, Katepwa and Neepawa, grown under dryland (rainfed) and irrigated conditions to determine physiological maturity (38% moisture content)

When we tested the application of the kernel probe with a moisture meter in the field, we found typical field dry-down moisture curves in wheat. The Fig. 3 shows the kernel moisture content at different dates in the four wheat varieties from early-dough onwards using HB300. The hexaploid bread wheat varieties, Katepwa and Neepawa, are known to have shorter days to maturity than the durum varieties, AAC Stronghold and Strongfield. Consequently Katepwa and Neepawa reached physiological maturity (38% moisture), as expected, earlier than AAC Stronghold and Strongfield. This pattern was seen under both dryland (rainfed) and irrigated conditions. With the grain filling and dry matter accumulation, water content continues to decline in a steady state (75–37%) in the kernel until it reaches physiological maturity (Calderini et al. [5]). Further kernel drying to the harvest maturity depends on the varietal genetics and several other environmental factors. Like other forms of moisture determination, the pin based kernel probe shows the dynamic change in moisture content in a kernel. But because the readings are instantaneous with a moisture meter, the researchers can start to collect more datapoints as the critical moisture approaches to better map a genotype for the variation in moisture.

The single kernel moisture probe differentiates the moisture content of the developing kernel in both irrigated and dryland environments (Fig. 3). This is important to determine the dry down period, which requires the information on the days to physiological maturity and harvest maturity in different growing environments (Calderini et al. [5]; Hanft and Wych [10]). The accuracy of the moisture content prediction will depend on the type and calibration ability of the moisture meters and a relationship with the oven dry method. Although, all devices showed good R^2^ value higher than 0.76, a moisture meter with calibration ability like HB300 is more accurate for predicting moisture content with high R^2^ closer to 1. This was true with both linear and non-linear regression analysis. It is likely that other moisture meters with option to calibrate as required would be equally useful when used with the pin probe. Additional studies will help to determine the broader application of pin probe with different moisture meters and in different grain crops.

No doubt, several improved technologies are available for grain moisture estimation, such as microwave resonators (Kraszewski et al. [15]) and NIR spectroscopy (Watanabe et al. [23]) the single kernel moisture device based on principles of electrical conductivity and resistance is relevant to wheat growers and industry personnel. Wheat growers can determine the moisture content in the field by sampling a few plants rather than transporting a combine and harvesting plants from a sizable area. They also would not have to deal with a sample of damp grain in their combine hopper, if they guessed wrong on the readiness of their crop to harvest. At industry level, determining right grain moisture at harvest will reduce the need for blending of high and low moisture grains to reduce the average moisture of stored grain lots thus maintaining the grain quality (Bonifacio-Maghirang et al. [2]). The single kernel probe is thus promising to alleviate such issues during harvest processing activities. The device could potentially be applied to other grain crops such as pea, chickpea, and beans, making it useful for broader application in research and at the farm. Further studies are required to help improve the device with more accuracy in measuring the kernel moisture and to determine the broader application in agriculture.

## Conclusions

In this paper, a proof of concept pin-based moisture probe was designed, fabricated and evaluated to measure moisture content of single wheat kernels. The probe works well with different commercial moisture readers. The device is suitable to track the moisture dynamics of the maturing kernels in the spike and to detect moisture based physiological maturity in a large population, such as breeding lines. It will be a useful tool for the wheat breeders and physiologists to study dry down in wheat, and allows farmers to determine the moisture content of their crops using a few randomly selected plant samples in the field. The probe design can be improved further for accuracy and simplicity for different grain crops.

## Materials and methods

All experiments were conducted from 2019 to 2024 in the field and laboratory at the Swift Current Research and Development Centre (Agriculture and Agri-Food Canada), Swift Current, SK. We used spikes collected from different wheat lines grown in the field and greenhouse for this study. The wheat lines were selected for different days to maturity to provide variable moisture levels in the grain. There were two parts to this study, one to adapt electronic moisture measuring devices to testing single wheat kernels with the subsequent comparison of those devices using a gravimetric measure of moisture. Secondly, by applying the best device from the first part of the study, proof of concept of using a single kernel moisture reader was performed for determining moisture based physiological maturity in wheat.

### Moisture meters

Since pin-based wood and corn moisture meters are well established and relatively inexpensive, this technology was chosen as a starting point for the development of a wheat single kernel moisture probe for assessing physiological maturity based on the kernel moisture content (38%). Moisture meters with pin-based probes were purchased from different commercial manufacturers and adjusted to a setting appropriate for grain [General Tools MMH800 (Setting-Wood), FLIR MR55 (Setting-Gr-9), Electrophysics Corn Probe MT808C (Setting-4), and KETT HB300 (Setting-none; user defined)] (Table 1). These meters measure direct current resistance whereby moisture act as an electrical conductor and solids such as wood fibers or corn kernel starchy endosperm act as an electrical insulator. The electrical resistance changes in direct proportion to moisture content and is calculated as a moisture percentage on the meter. Pin-less moisture meters based on electromagnetic conduction were used in a preliminary study but were discontinued due to high variability in measurements. The moisture detection capability of different pin-based moisture devices ranged from 0- to100% and varied with inbuilt capability of these devices (Table 1).

### Designing a pin-based moisture probe

The pin probes of commercial wood and corn moisture meters were much too large and spaced too far apart to be useable to measure individual wheat kernels. For application in wheat, much narrower spaced pins were required. We developed probe prototypes, connectable to each moisture meter, designed with heavy duty stainless steel denim needles spaced approximately 2.5 mm apart. The heavy duty stainless steel needles provided a good balance of high electrical conductivity while maintaining the tensile strength needed to pierce the glume through to the wheat kernel seed coat in a small enough form factor so as not to damage overall kernel or spikelet structure. Pin distance was maintained by pushing pins through the center of a #00 rubber stopper maintaining the 2.5 mm separation. Each pin emerged 20 mm from narrow end of the stopper to form the probe head. Two 1.2 m lengths of #22 AWG wire leads were then soldered to the dull ends of the pins protruding from the wide end of the stopper. The wires were fed through a plastic tubular body and the one end of this tube body was epoxied to the rubber stopper to form a pen-style probe. Heat shrink tubing was then applied to the assembly for additional union strength and to secure the wiring emerging from the bottom of the probe body. Lastly, the wire ends were soldered to a suitable connection end depending on the moisture meter connection (BNC, RCA or terminal ring) (Fig. 2). The functionality and accuracy of the sensor probe was verified with a commercially available moisture calibration discs for 16% moisture content.

### Preparing kernel samples for moisture detection

Spikes for the kernel moisture study were collected from the field starting at the milk dough stage up to the maturity stage (∽ 18% moisture) when the grain was hard enough to still pierce with the probe pins. Spikes from randomly selected plants from wheat field plots were cut from the peduncle and brought to the laboratory in a sealed plastic bag to avoid moisture loss. A set of minimum 100 kernels were used for moisture measurement for each experimental batch.

### Measuring kernel moisture content

Kernel moisture was measured with two different methods described below.

#### Moisture content using digital moisture meters

Immediately after arriving in the laboratory, kernels from the middle portion of the spike were removed from the spikelet using forceps and weighed for fresh weight before measuring moisture content. The moisture content in the kernel was tested using all four moisture meters connected with the pin probe. The moisture meters were set at specific modes following the user’s manual, with the exception of HB300 that was calibrated for wheat kernel using a setting at 22 °C. The pins of the probe connected with the moisture meter were pushed into the kernel longitudinally and the reading on the moisture meter representing the moisture content present in the kernel was recorded. Immediately after recording the moisture content with each moisture reader, the kernel samples were transferred to an oven for complete drying using oven drying method.

#### Gravimetric oven dry method

The kernel samples were weighed on a micro weighing balance, with 0.0001 g sensitivity, for fresh weight and placed individually in a size 3 paper envelop to avoid humidity buildup. The samples were dried in an oven with circulating air at 130 °C for 19 h to a minimum, but no longer than 76 h depending operational limitations (Fisher Scientific, US). After drying, the kernel samples were weighed again for dry weight data and the reference moisture value was calculated as follows:

Kernel moisture % = ([wet kernel mass - dry kernel mass]/wet kernel mass) × 100.

Probe based moisture meter readings and reference kernel moisture readings by the oven dry method as percent moisture were compared for interpreting the accuracy of the method.

### Determining kernel moisture based physiological maturity

Wheat varieties differ in reaching the harvest maturity depending on the genetics and the environmental factors. In a field trial, four wheat varieties including two hexaploid bread wheat class (Neepawa, Katepwa) and two in the durum pasta wheat class (Strongfield, AAC Stronghold) were used for moisture dynamics and determining moisture (38%) based physiological maturity. To create further variability in maturity of wheat varieties under different environments, we used irrigated and rainfed field conditions. The pin-probe connected with the HB300 moisture device was used to detect moisture of individual developing kernels by piercing the spikelet through the glume and noting the moisture content. The plants in the plots were also assessed and documented for grain development, change in green color of the glume and rachis, and loss of moisture in the kernel. The kernel hardness was checked regularly for thumb nail impression to determine maturity. In addition to using a value around 38% moisture content, visual observations were used to support the determination of physiological maturity (Calderini et al. [5]).

### Statistical analysis

Moisture meter data were collected from two independent experiments and a minimum of 100 randomly collected kernels were used in each experiment. Each experiment was repeated three times. Field data were collected from a single experiment with three replicated plots, and three randomly selected spikes per plot were evaluated for each variety in the study. The moisture data were subjected to analysis of variance, correlation, and regression analysis using JMP 17.0 (SAS, USA). The data collected with moisture meters and the oven dry method were analysed using both linear regression and non-linear regression (2nd degree polynomial). Moisture meter data were used as independent variable and oven moisture data as dependent variable. Coefficient of determination (R^2^) were calculated to determine the accuracy of the moisture meters using single kernel probes. The Pearson’s and Spearman’s coefficients of correlation (r) were calculated for the linear and non-linear relationship, respectively. Standard error of performance/prediction (SEP) were calculated using the formula reported by Zhang et al. [24]. Data were plotted using Excel programme in MS Office using means and standard errors.

## Data Availability

All the data reported in the study is analysed and presented in tables and figures. Additional datasets used in the current study are available from the corresponding author in accordance with the data sharing policies of Agriculture and Agri-Food Canada.
